# Compound but non-linked heterozygous p.W14X and p.L279 V LPL gene mutations in a Chinese patient with long-term severe hypertriglyceridemia and recurrent acute pancreatitis

**DOI:** 10.1186/s12944-018-0789-2

**Published:** 2018-06-19

**Authors:** Xiaoyao Li, Qi Yang, Xiaolei Shi, Weiwei Chen, Na Pu, Weiqin Li, Jieshou Li

**Affiliations:** 1 Surgical Intensive Care Unit (SICU), Department of General Surgery, Jinling Hospital, Medical School of Nanjing University, Nanjing, China; 2 Department of Gastroenterology, Clinical Medical College, Yangzhou University, Yangzhou, Jiangsu China

**Keywords:** Hypertriglyceridemia, Lipoprotein lipase, Mutation, Acute pancreatitis, LPL gene

## Abstract

**Background:**

Variants in the lipoprotein lipase (LPL), apolipoprotein C-II (APOC2), apolipoprotein A-V (APOA5), GPIHBP1 and LMF1 genes may cause severe hypertriglyceridemia (HTG), which is now the second-leading aetiology of acute pancreatitis in China.

**Methods:**

The patient and his family were assessed for gene variants by Sanger sequencing of exons and exon-intron junctions of the LPL, GPIHBP1, APOA5, APOC2, and LMF1 genes. Post-heparin blood was collected for LPL mass and activity detection.

**Results:**

The patient had suffered from long-term severe hypertriglyceridemia and recurrent abdominal pain for over 30 years, since age 26, and 3 bouts of acute pancreatitis. Two heterozygous LPL single-nucleotide polymorphisms (SNPs) were compound but dislinked: a single-nucleotide substitution (c.42G > A) resulting in the substitution of tryptophan with a stop codon (p.W14X) in one allele, and a single-nucleotide substitution (c.835C > G) resulting in a leucine-to-valine substitution (p.L279 V) in another allele. Only one SNP, p.L279 V, was detected in his son. Post-heparin LPL activity and mass were also lower in the patient.

**Conclusion:**

Two heterozygous LPL SNPs, W14X and L279 V, were newly found to be compound but dislinked, which may cause long-term severe hypertriglyceridemia and recurrent acute pancreatitis.

## Background

Hypertriglyceridemia (HTG), especially severe hypertriglyceridemia (sHTG, serum triglycerides more than 11.3 mmol/L), may cause hepatosplenomegaly, stomach ache, lipaemia retinalis, and eruptive xanthomas and is a high-risk factor for recurrent acute pancreatitis (AP) [1–4]. HTG can be divided into primary and secondary HTG by its aetiology. [5] Primary HTG is mainly caused by genetic loss of function, including of lipoprotein lipase (LPL), which catabolizes triglycerides (TG) in non-hepatic tissues [6, 7]; apolipoprotein C-II (APOC2), which acts as an essential LPL activator; apolipoprotein A-V (APOA5), which stabilizes the lipoprotein–LPL complex; lipase maturation factor 1 (LMF1), which modulates the folding and expression of LPL; or glycosylphosphatidylinositol-anchored high-density lipoprotein-binding protein 1 (GPIHBP1), which mediates the transmembrane transport of LPL and binding between lipoprotein and LPL [8, 9]. Secondary HTG is caused by obesity, poorly controlled diabetes, excessive alcohol consumption, hypothyroidism, nonalcoholic fatty-liver disorder, renal failure, pregnancy, nephrotic syndrome, drugs or rare metabolic disorders [8, 10, 11]. Gene variants of LPL are reported to be the most usual cause of primary sHTG [12].

LPL belongs to the same lipase superfamily that includes hepatic lipase (HL), endothelial lipase (EL) and pancreatic lipase [13]. As a homodimeric glycoprotein, LPL is mainly synthesized in the parenchymal cells of the heart, skeletal muscle and adipose tissues. Once secreted, it is transported across the luminal surface and anchors to the vascular endothelial cells by heparan sulphate proteoglycans, where it hydrolyses triglyceride in chylomicrons (CM) and very low-density lipoproteins (VLDL) [9]. The LPL gene is encoded by 10 exons, spans approximately 30 kb on chromosome 8p22, and encodes a protein with 475 amino acids [9]. Genetic loss of function can be caused by gene mutations in different forms, such as insertion, duplication, deletion, nonsense mutations, frameshift mutations, and missense mutations. To date, more than 200 LPL mutations or single-nucleotide polymorphisms (SNPs) have been reported to cause severe hypertriglyceridemia syndrome and recurrent acute pancreatitis. Additionally, several heterozygous LPL SNPs are compound and cause various clinical features as severe as those caused by homozygous SNPs [14–16]. Here, we report two heterozygous LPL SNPs, W14X and L279 V, in a male Chinese patient, which were found be compound but dislinked. As dietary fat intake significantly modifies serum lipids [17], the patient’s TG level has dropped significantly since changing to a completely vegan diet.

## Methods

### Subjects

The proband was a 61-year-old Chinese male attending Jinling Hospital in May 2017 to check his recovery from previous acute pancreatitis. He had long-term severe hypertriglyceridemia, recurrent abdominal pain for over 30 years, and 3 bouts of recurrent acute pancreatitis. Since he was 21 years old (1977), he had suffered recurrent abdominal pain with no obvious basis. Since the age of 26 (1982), his serum triglyceride level had been markedly elevated to a level that could be defined as severe hypertriglyceridemia (> 11.3 mmol/L) [18]. In 2010, when he was 54 years old, type II diabetes was diagnosed, and he has taken metformin ever since. Meanwhile, he has had 3 bouts of recurrent acute pancreatitis, when he was 29 (1985), 55 (2011) and 60 years old (2016).

The first attack of acute pancreatitis, in 1985, was diagnosed as involving partial pancreatic necrosis and was treated by laparotomy. His second and third acute pancreatitis episodes happened in 2011 and 2016, respectively, and were conservatively treated by fasting, inhibition of enzymes, treatment for infection, and nutrition therapy.

The patient has followed a low-fat, high-protein, high-carbohydrate diet since 1985. After the DM diagnosis, he changed to a low-fat, low-carbohydrate, and high-protein diet. He even changed to a vegan diet in Nov. 2017. He had no drinking or smoking history and a low BMI of 16.8, and has taken fenofibrate regularly since 2015; however, recurrent abdominal pain was still occurring with no obvious cause.

Here, we sequenced the LPL, APOC2, APOA5, LMF1 and GPIHBP1 genes in the proband, his son and his wife. This study was approved by the Ethics Committee of Jinling Hospital, and signed informed consent was obtained from all subjects.

### Serum lipid profile analysis

After obtaining informed written consent for the clinical and genetic study, blood samples were taken from the patient after fasting for 12 h. Serum TG, total cholesterol (TC), high-density lipoprotein (HDL), and low-density lipoprotein (LDL) were measured enzymatically on an automatic analyser (Hitachi High-Tech, 7600–120, Japan). Serum lipoproteins, including apolipoprotein A (APO-A) and apolipoprotein B (APO-B), were measured by immunoturbidimetric assays on an automatic analyser (Hitachi High-Tech, 7600–120, Japan).

### Measurement of post-heparin LPL mass and activity

Blood samples were collected into Na-EDTA tubes 10 min after an intravenous heparin (60 IU/kg body weight) injection. Plasma LPL protein mass concentration was determined by immunoassay using a Human LPL Elisa kit (TSZ Biological Trade, USA). Values based on a standard curve were acquired in parallel assays. LPL activity in the post-heparin plasma was determined using an LPL activity assay kit (MAK109, SIGMA, USA).

### Polymerase chain reaction (PCR) amplification and DNA sequencing of candidate genes

Genomic DNA was extracted from the peripheral blood cells using a Gentra Puregene Blood kit (Qiagen, Dusseldorf, Germany) according to the manufacturer’s instructions. Candidate genes involved in serum TG metabolism, including LPL, APOA5, APOC2, LMF1 and GPIHBP1, were amplified by PCR using primer sequences. Individual exons with flanking intron sequences of five genes were amplified. The PCR products were sequenced by Sanger sequencing. One nonsense change and one missense change were identified in the LPL gene. The variants were confirmed by repeated sequencing (3 times) to verify the results.

### Species examination

We examined the evolutionary conservation of the W14 and L279 amino acids across various species, from chimpanzee (a close evolutionary relative) to mallard and zebra fish (both distant evolutionary relatives).

## Results

### Serum lipid and lipoproteins

The proband had long-term hypertriglyceridemia and had several fasting serum TG measurements on record since 1982. After first checking his recovery in our center in May 2017, we measured his fasting blood glucose (Glu) and 6 lipoproteins, including TG, TC, HDL, LDL, APO-A and APO-B, every month. In 1982, when first diagnosed with HTG, his TG was 11.9 mmol/L (1054.7 mg/dL). In 1985, at the first onset of acute pancreatitis, his TG was 33.8 mmol/L (2992.8 mg/dL). In 1988, 1989, 1991 and 1993, the proband had fasting TG of 25.3 mmol/L (2243.3 mg/dL), 18.3 mmol/L (1624 mg/dL), 16.7 mmol/L (1478.8 mg/dL), and 18.9 mmol/L (1673.5 mg/dL), respectively, which indicated long-term severe hypertriglyceridemia. The patient used no medicine or other treatment and continued his low-fat, high-carbohydrate, and high-protein diet.

In 2010, the proband was diagnosed with type II diabetes mellitus (DM) and changed his diet to low fat, low carbohydrate, and high protein. However, still he had HTG and another attack of acute pancreatitis in 2011, with a high fasting TG level of 38.6 mmol/L (3417.8 mg/dL). Then, since 2015, the patient has regularly taken fenofibrate at a dosage of one pill per day, but still suffered from HTG and had a third bout of recurrent acute pancreatitis. At the onset of AP, his serum TG level was 34.16 mmol/L (3024.7 mg/dL). After 5 days of fasting and therapy, the TG level dropped to 4.05 mmol/L (358.6 mg/dL).

**Fig. 1:**
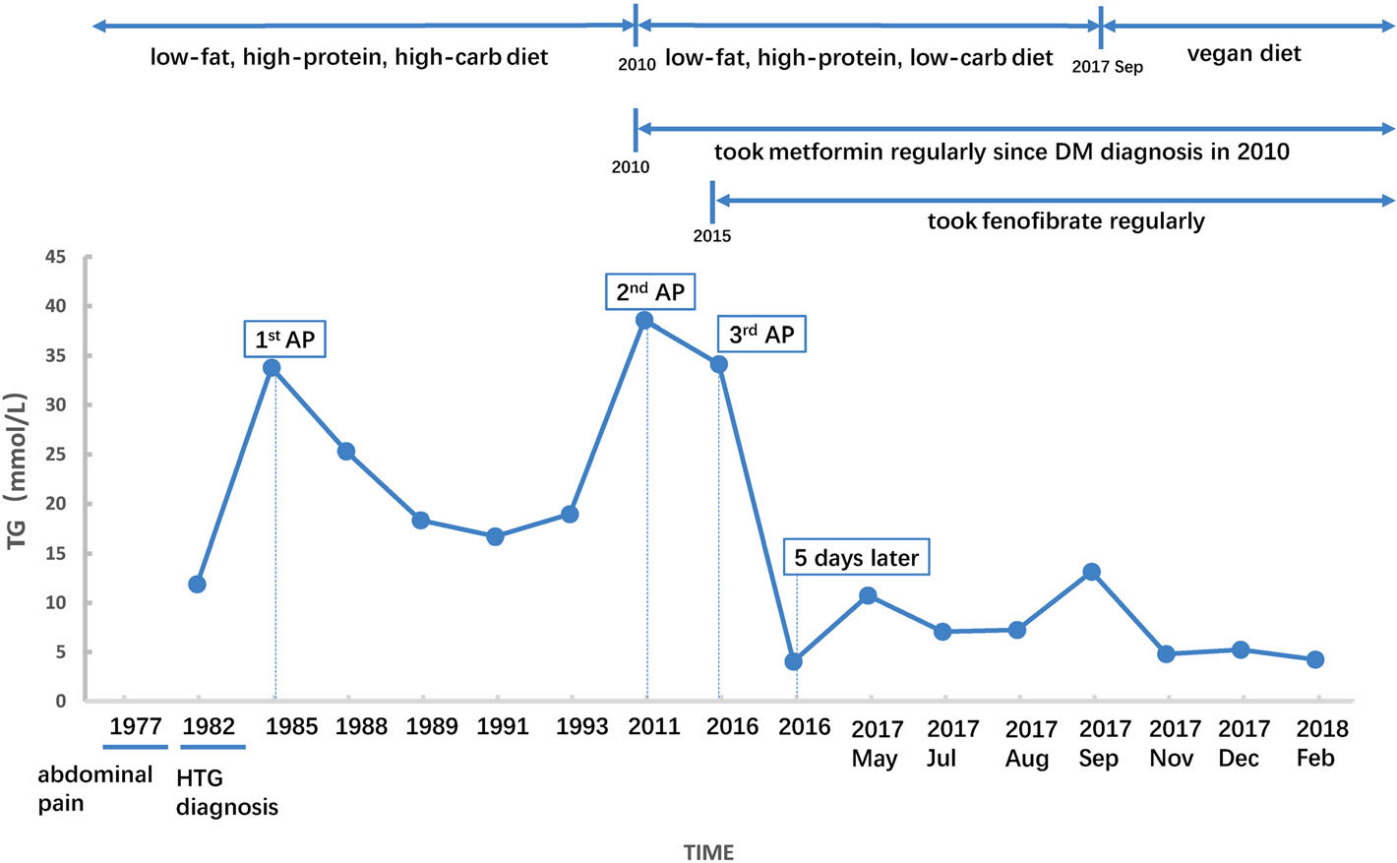
Details of TG levels and disease time points of the proband TG: triglycerides; AP: acute pancreatitis

Recently, in May 2017, the patient came to our center, the largest AP transfer and therapy center in China, to check his recovery from acute pancreatitis. We measured his fasting serum Glu, TG, TC, HDL, LDL, APO-A, and APO-B monthly. In May, July, August, and September 2017, the patient still suffered HTG, with fasting TG levels, respectively, of 10.75 mmol/L (951.9 mg/dL), 7.08 mmol/L (626.9 mg/dL), 7.26 mmol/L (642.8 mg/dL), and 13.17 mmol/L (1166.1 mg/dL). After the TG measurement of 13.17 mmol/L in September 2017, the patient changed to a completely vegan diet. At his last update, the patient had a remarkable and consistent reduction in TG and even could be defined as having moderate hypertriglyceridemia [18]. In November and December 2017 and February 2018, his fasting serum TG levels, respectively, were 4.81 mmol/L (425.9 mg/dL), 5.25 mmol/L (464.9 mg/dL), and 4.25 mmol/L (376.3 mg/dL). The significant drop in the TG also reduces his risk of AP, as the multivariable adjusted HR for acute pancreatitis was 1.17 per 1 mmol/L higher triglycerides in one study [19], and he has not had AP for approximately 2 years. In the nine-month follow-up detection period, although the TG fluctuated from 13.17 to 4.25 mmol/L, the other 5 lipoproteins were consistently in the normal ranges. The detailed TG level and the disease time point of the proband are shown in Fig. 1, and the follow-up measurement of Glu and 6 lipoproteins are shown in Table 1.

**Table 1:** Lipid laboratory profiles of the proband at different follow-up times

	TG (mmol/L)	TC (mmol/L)	HDL (mmol/L)	LDL (mmol/L)	APO-A (g/L)	APO-B (g/L)	Glu (mmol/L)
2017 May	10.75	5.93	1.02	0.71	NA	NA	5.61
2017 Jul	7.08	3.99	0.99	1.37	1.23	0.66	6.31
2017 Aug	7.26	3.82	0.9	1.23	1.04	0.75	8.09
2017 Sep	13.17	4.77	1.36	1.41	1.09	0.7	6.89
2017 Nov	4.81	3.34	2.4	1.23	1.8	1.05	5.96
2017 Dec	5.25	3.75	0.83	1.33	1.23	0.71	6.71
2018 Feb	4.25	3.75	0.78	1.39	0.98	0.64	7.38

*TG* triglycerides; *TC* total cholesterol; *HDL* high-density lipoprotein; *LDL* low-density lipoprotein; *APO-A* apo-lipoprotein A; *APO-B* apo-lipoprotein B; *Glu* glucose

His son and wife had normal serum TG, 1.3 mmol/L and 0.7 mmol/L, respectively. They are aged 35 and 60 years now.

### Genetic and biochemical analysis

**Fig. 2:**
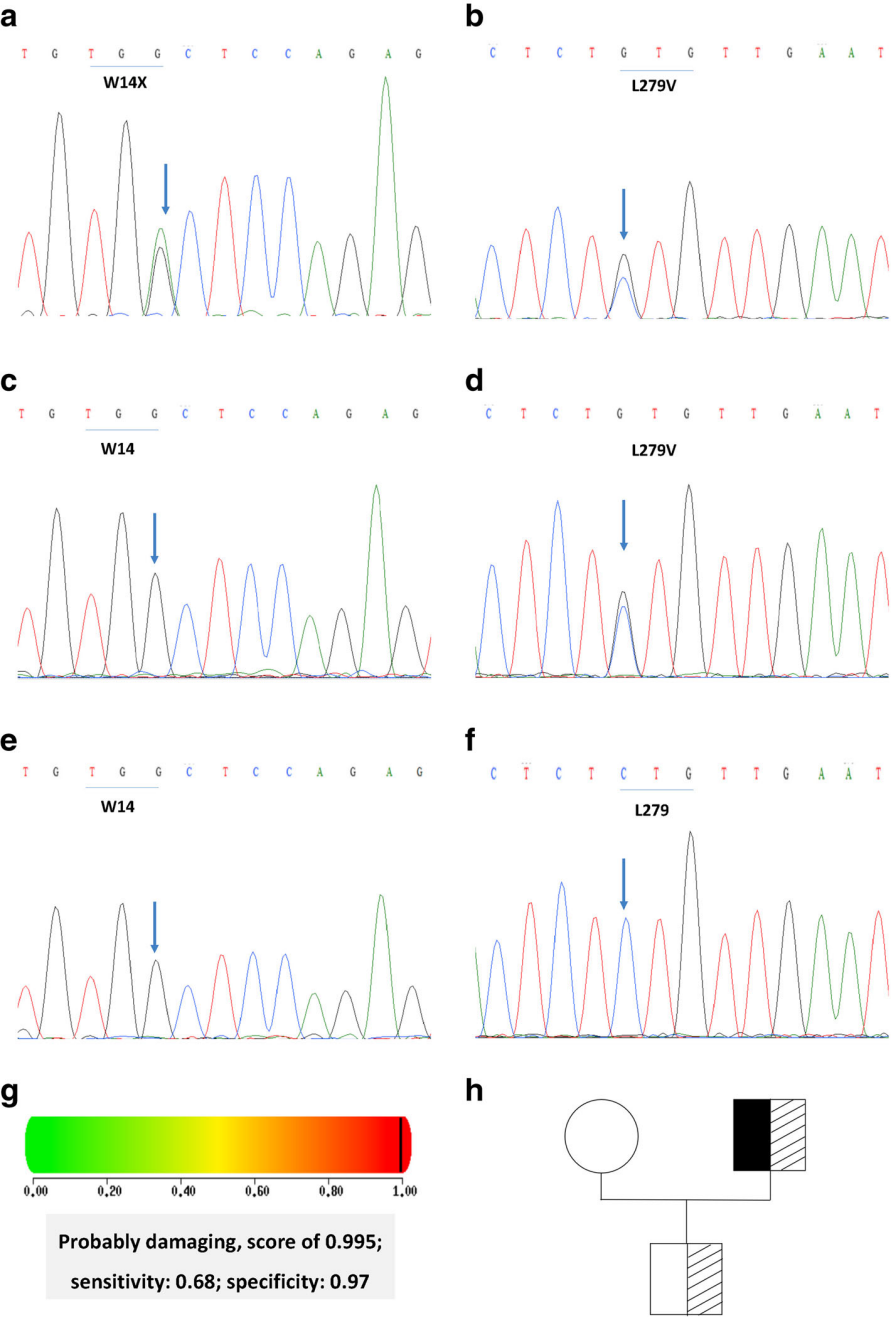
Partial nucleotide sequences of the lipoprotein lipase (LPL) gene and pedigree of the family. **a** The proband was heterozygous for the nucleotide substitution in exon 1 that resulted in W14X. The arrow shows the nucleotide substitution from G to A. **b** The proband was heterozygous for the nucleotide substitution in exon 6 that resulted in L279 V. The arrow shows the nucleotide substitution from C to G. **c** W14X was not detected in the son of the proband. **d** The son of the proband was heterozygous for the nucleotide substitution in exon 6, which resulted in L279 V. The arrow shows the nucleotide substitution from C to G. **e** W14X was not detected in the wife of the proband. **f** L279 V was not detected in the wife of the proband. **g** L279 V was predicted to be damaging by polyphen software. **h** Pedigree of the family. Black boxes represent W14X, and dashed boxes represent L279 V

**Fig. 3:**
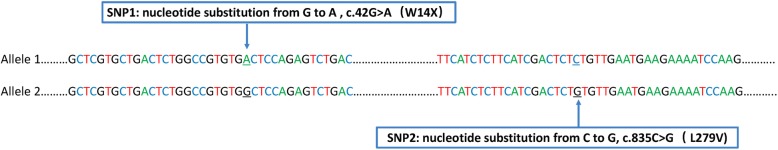
Diagrammatic picture of two non-linked but compound heterozygous SNPs, W14X and L279 V

Two heterozygous SNPs in the LPL gene were identified in the proband, a single-nucleotide substitution (c.42G > A) resulting in the substitution of 14Trp (TGG) with a stop codon (TGA) (p.W14X) in exon 1, and a nucleotide substitution (c.835C > G) resulting in a leucine-to-valine substitution (p.L279 V) in exon 6 (Fig. 2a-f). The two SNPs were newly found to be compound and, here, were named SNP1 (p.W14X) and SNP2 (p.L279 V). These two SNPs were more than 10,000 nucleotides from each other, which made it difficult to analyze their linkage relationship. Instead, we examined the genes of the proband’s son and his wife. Only SNP2 (p.L279 V) was detected in his son, and neither in his wife. The results showed that SNP1 and SNP2 were in different alleles, which indicated these two SNPs were compound but dislinked (Fig. 3).

**Fig. 4:**
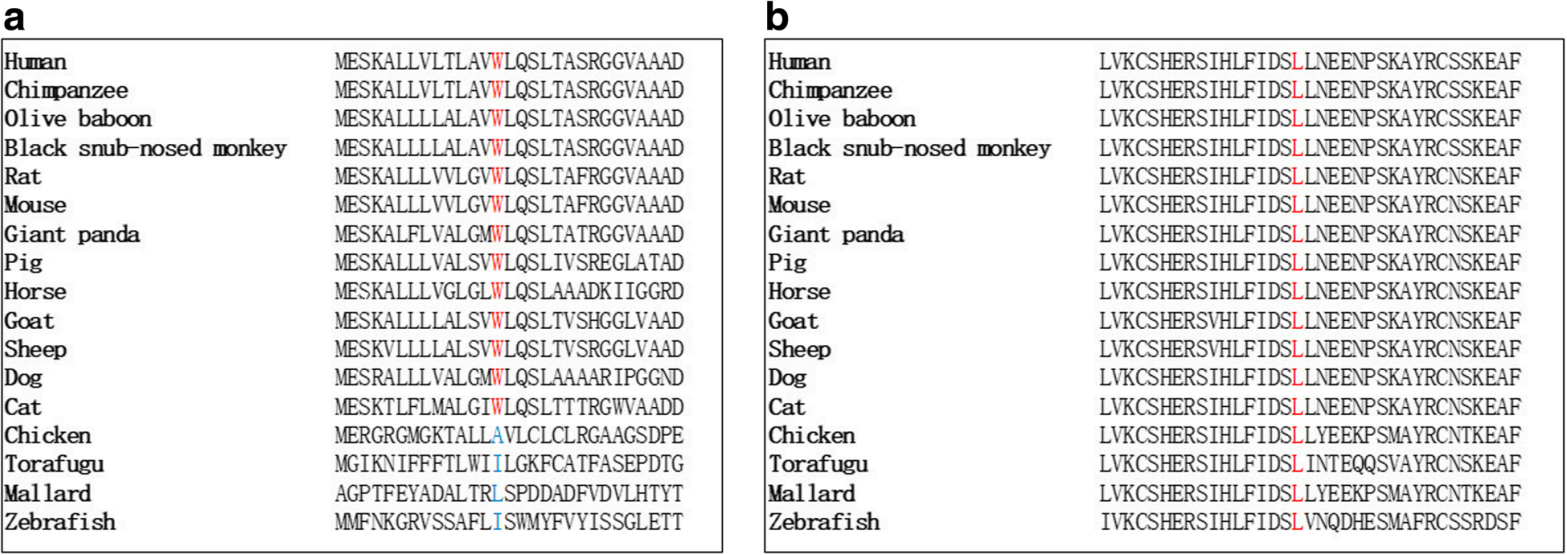
Evolutionary conservation of the W14 (**a**) and L279 (**b**) amino acid residues in various species

SNP1, p.W14X, directly caused LPL loss of function, due to the shortening of the LPL protein from 475 to 14 amino acids. SNP2, p.L279 V, which is registered as rs371282890 in NCBI’s SNP database (https://www.ncbi.nlm.nih.gov/SNP/snp_ref.cgi?rs=371282890), has been reported to be pathogenic by PANTHER, SIFT, and SNPs3D. [5] Here, we used another software program, polyphen (http://genetics.bwh.harvard.edu/pph2/), to analyze the pathogenicity score of p.L279 V. The results showed it was probably damaging, with a score of 0.995 (Fig. 2g). No mutations were found in the APOC2, APOA5, GPIHBP1 or LMF1 genes. The original Sanger sequencing results, polyphen score, and pedigree of the family are shown in (Fig. 2h) Moreover, both W14 and L279 were highly conserved across the species (Fig. 4), suggesting that the two amino acids may play important roles in LPL maturity and/or function.

### Post-heparin plasma LPL activity

We analyzed LPL activity and mass in the post-heparin plasma of the patient in November 2017, when his TG was 4.81 mmol/L. LPL mass was 185.7 U/L, considerably lower than the mean value of normal controls (382 U/L, *n* = 10). LPL activity was 7.7 nmol/mL, which was significantly lower than the mean value of normal controls (19.3 nmol/mL, *n* = 10). LPL mass was 282.7 U/L and LPL activity was 16.8 nmol/mL in his son, while LPL mass was 380.6 U/L and LPL activity was 18.8 nmol/mL in his wife, which were both near the mean values of normal controls.

## Discussion

In this study, we present a male Chinese patient who manifested long-term severe hypertriglyceridemia, recurrent abdominal pain and 3 bouts of acute pancreatitis. The sequencing results showed the proband had two heterozygous SNPs in the LPL gene, W14X and L279 V, which were newly found to be compound but dislinked. No mutations in APOA5, APOC2, LMF1 or GPIHBP1 were detected. His son was heterozygous for L279 V but had no consistent abdominal pain, HTG, acute pancreatitis, or other disease history.

LPL is the rate-limiting enzyme for the hydrolysis of the TG core of circulating TG-rich lipoproteins (CM and VLDL), which are produced by many tissues, including adipose tissue, cardiac and skeletal muscle, islets, and macrophages [20]. Deficient LPL could dramatically increase CM levels and become a high-risk factor for acute pancreatitis, hypertriglyceridemia, diabetes mellitus, and other metabolic disorders [21, 22]. The W14X SNP in LPL was first reported in a Japanese woman with hypertriglyceridemia. She had a homozygous W14X variation, resulting in nonsense-mediated decay, and her post-heparin plasma showed no LPL protein or activity. The daughters of the proband were heterozygous for this variant, but they presented with normal serum TG and normal LPL protein levels and activity in post-heparin plasma [23].

The other SNP, L279 V, has been frequently reported and well-studied. The first case report of L279 V was a heterozygous Hong Kong Chinese patient reported by Chan L et al [11]. Then, some population studies reported that the frequency of L279 V varied from 1/160 to 5/101 in Asian countries, such as Thailand, Mainland China, Taiwan and Hong Kong [5, 11, 12, 24–27]. But in European continental ancestry groups, L279 V has never been reported, which may suggest that genetic variations in LPL differ between different races and ethnic backgrounds. L279 V is a disease-causing missense change with LPL deficiency only among Asian patients, probably because of the founder effect [5, 24]. L279 V has been verified to be pathogenic by both biomedical methods and in vitro studies, such as transfecting wild-type and L279 V LPL plasmids into COS-1 cells [11, 27]. However, there has been no in vivo research to analyze its particular pathogenic mechanism. L279 V, located in exon 6 of the LPL gene, acts as a heparin binding site using two structurally relevant disulphide bridges (Cys278-Cys283 and Cys264-Cys275). Leucine 279 may play a significant role in the catalytic function of LPL by constituting one of the two disulphide bridges (Cys278-Cys283), which are important for the catalytic function of heparin binding [12, 27].

Furthermore, we assessed the W14 and L279 residues by multiple-species sequence alignment. The results showed that W14 and L279 were both highly conserved throughout evolution, from chimpanzee to torafugu and zebra fish, suggesting their important roles in LPL function.

Ageing, obesity, diet, drugs, hyperinsulinaemia, exercise and other factors may promote clinical manifestations of LPL-gene heterozygous defects [23]. Goni et al. found that a low-fat diet improved the lipid profile of MTNR1B rs10830963 genetic variants, indicating the significance of personalized dietary interventions in improving lipid metabolism caused by genetic variation [17]. Our male Chinese patient has been taking fenofibrate regularly since 2015, but his serum TG was still above 7 mmol/L. However, since he changed to a vegan diet in November 2017, his TG decreased to approximately 5 mmol/L, without any changes in drug use or exercise frequency. His 35-year-old son, with one heterozygous SNP (L279 V), has been maintaining a healthy lifestyle including a low-fat diet and high exercise frequency and intensity, which may help him to maintain normal levels of TG and LPL activity [28].

The genetic basis of most severe hypertriglyceridemia and recurrent pancreatitis patients is still unknown and remain a subject of further investigations. In future research, we aim to detect the rates of the W14X and L279 V mutations in a large Chinese population and verify the pathogenic mechanisms of these two SNPs in vivo.

## Conclusion

Here, we present a typical male Chinese patient with long-term hypertriglyceridemia, recurrent abdominal pain, and recurrent pancreatitis. By Sanger sequencing of the LPL, APOA5, APOC2, LMF1, and GPIHBP1 genes, two LPL SNPs, W14X and L279 V, were newly found to be compound but dislinked in the proband. The proband’s son had one SNP, L279 V, and normal TG, which may be due to his healthy lifestyle including a low-fat diet and high intensity and frequency of exercise. Therefore, we propose that it is important for HTG patients to have both early-phase gene detection and a healthy lifestyle.

## Abbreviations

AP: Acute pancreatitis
APO-A: Apo-protein A
APOA5: Apolipoprotein A-V
APO-B: Apo-protein B
APOC2: Apolipoprotein C-II
BMI: Body mass index
Carb: Carbohydrate
CM: Chylomicron
DM: Diabetes mellitus
EL: Endothelial lipase
Glu: Glucose
GPIHBP1: Glycosylphosphatidylinositol-anchored high-density lipoprotein binding protein 1
HDL: High density lipoprotein
HL: Hepatic lipase
HTG: Hypertriglyceridemia
LDL: Low density lipoprotein
LMF1: Lipase maturation factor 1
LPL: Lipoprotein lipase
PCR: Polymerase chain reaction
sHTG: Severe hypertriglyceridemia
SNP: Single nucleotide polymorphism
SNP1: p.W14X
SNP2: p.L279 V
TC: Total cholesterol
TG: Triglyceridemia
